# Prenatal Exposure to a Maternal High Fat Diet Increases Hepatic Cholesterol Accumulation in Intrauterine Growth Restricted Rats in Part Through MicroRNA-122 Inhibition of Cyp7a1

**DOI:** 10.3389/fphys.2018.00645

**Published:** 2018-05-29

**Authors:** Erin K. Zinkhan, Baifeng Yu, Amnon Schlegel

**Affiliations:** ^1^Department of Pediatrics, Division of Neonatology, University of Utah School of Medicine, Salt Lake City, UT, United States; ^2^University of Utah Molecular Medicine Program (U2M2), University of Utah School of Medicine, Salt Lake City, UT, United States; ^3^Department of Internal Medicine, Division of Endocrinology, Metabolism and Diabetes, University of Utah School of Medicine, Salt Lake City, UT, United States; ^4^Department of Biochemistry, University of Utah School of Medicine, Salt Lake City, UT, United States; ^5^Department of Nutrition and Integrative Physiology, College of Health, University of Utah, Salt Lake City, UT, United States

**Keywords:** intrauterine growth restriction, high fat diet, microRNA-122, cholesterol, cholesterol 7α hydroxylase, perinatal environment, fetal origins of adult disease

## Abstract

Intrauterine growth restriction (IUGR) and consumption of a high saturated fat diet (HFD) increase the risk of hypercholesterolemia, a leading cause of morbidity and mortality. The mechanism through which the cumulative impact of IUGR and in utero exposure to a maternal HFD increase cholesterol levels remains unknown. Cholesterol 7α hydroxylase (Cyp7a1) initiates catabolism of cholesterol to bile acids for elimination from the body, and is regulated by microRNA-122 (miR-122). We hypothesized that IUGR rats exposed to a maternal HFD would have increased cholesterol and decreased Cyp7a1 protein levels in juvenile rats, findings which would be normalized by administration of a miR-122 inhibitor. To test our hypothesis we used a rat model of surgically induced IUGR and fed the dams a regular diet or a HFD from prior to conception through lactation. At the time of weaning, IUGR female rats exposed to a maternal HFD had increased hepatic cholesterol, decreased hepatic Cyp7a1 protein and hepatic bile acids, and increased hepatic miR-122 compared to non-IUGR rats exposed to the same HFD. *In vivo* inhibition of miR-122 increased hepatic Cyp7a1 protein and decreased hepatic cholesterol. Our findings suggest that IUGR combined with a maternal HFD decreased cholesterol catabolism to bile acids, in part, via miR-122 inhibition of Cyp7a1.

## Introduction

Intrauterine growth restriction (IUGR) results from inadequate fetal nutrition during gestation and increases the risk of hypercholesterolemia in adulthood (Forsdahl, 1978; Barker et al., 1989, 1993). IUGR individuals that consume a high fat diet have higher serum cholesterol levels compared to non-IUGR individuals also consuming a high fat diet (Robinson et al., 2006). Thus IUGR may increase the susceptibility of an individual to high fat diet-induced hypercholesterolemia. The average American, including women during pregnancy, consumes approximately 23–33 g of saturated fat and up to 400 mg cholesterol daily, both of which exceed the recommended fat and cholesterol intake of approximately 16 g saturated fat and 200 mg cholesterol (Wright et al., 2003; Reynolds et al., 2013). Thus IUGR infants can be exposed in utero to a maternal high fat diet.

The liver contributes significantly to the regulation of serum cholesterol levels. The liver regulates hepatic cholesterol levels through multiple pathways. One of these pathways involves catabolism of cholesterol to bile acids and excretion from the body via cholesterol 7 alpha-hydroxylase (Cyp7a1). Cyp7a1 is regulated by the oxysterol-binding transcription factor Liver X Receptor α (Lxrα). In addition to transcriptional regulation of Cyp7a1 by transcription factor Lxrα, Cyp7a1 is also regulated by microRNA-122 (miR-122), a small RNA that destabilizes Cyp7a1 mRNA thus decreasing Cyp7a1 translation and bile acid synthesis (Song et al., 2010). Other pathways regulating liver sterol metabolism include the following: transcriptional regulation of *de novo* cholesterol production by Sterol regulatory element binding protein 2 (Srebp2), multistep regulation of the rate-limiting enzyme of sterol synthesis 3-hydroxy-3-methylglutaryl-CoA reductase (Hmgcr), import of cholesterol from the plasma via the low density lipoprotein (LDL) receptor (Ldlr), export of high density lipoprotein (HDL) cholesterol to the plasma via ATP binding cassette transporters g1 (Abcg1) and Abca1, and export of very-low density lipoprotein (VLDL) cholesterol to the plasma by Fatty acid synthase (Fasn) and Microsomal transferase protein (Mtp).

We previously demonstrated that adult IUGR rats fed a HFD from weaning through adulthood had increased cholesterol, decreased Cyp7a1 protein, and decreased hepatic bile acids (Zinkhan et al., 2014). Using a rat model of surgically induced IUGR and in utero exposure to maternal HFD consumption, we hypothesized that weanling IUGR rats exposed to a maternal HFD in utero would have increased cholesterol, decreased Cyp7a1 protein, increased hepatic bile acids, and increased miR-122 compared to non-IUGR rats exposed to the same maternal HFD. Further, we hypothesized that inhibiting miR-122 would normalize the IUGR- and maternal HFD-induced increase in cholesterol and decrease in Cyp7a1 protein.

## Materials and Methods

### Animal Husbandry and Study Design

This study was carried out in accordance with the recommendations of the University of Utah Animal Care Committee. The protocol was approved by the University of Utah Animal Care Committee. Male and non-pregnant female 50-day old Sprague Dawley rats were obtained from Charles River Laboratories, Inc. (Wilmington, MA, United States). Male rats used for mating were kept on a regular rat chow (Reg, Harlan-Teklad, TD.8640, Madison, WI) throughout the study. Non-pregnant female rats were placed either on a regular rat chow (TD.8640) or a high fat diet rat chow (Harlan Teklad, TD.110526) for 5 weeks prior to mating through the end of lactation on postnatal day (P) 21. The regular chow contained 17% kcals from fat, 54% kcals carbohydrate, and 29% kcals from protein. Soybean oil was the fat source in the regular diet at 60 g/kg food, and the regular diet contained 0.03% w.w cholesterol. The HFD contained 44% kcals from fat, 40% kcals from carbohydrate, and 16% kcals from protein. A mixture of soybean oil at 10 g/kg and milk fat comprised the fat source in the HFD, for a total of 65% saturated fat. The HFD also contained 1% w.w cholesterol and 0.5% cholic acid to aid in fat absorption. The protein content of the HFD was lower than the protein content of the regular diet. The protein content of the HFD was chosen to be similar to protein consumption in the United States today (Wright et al., 2003) and was sufficient for normal mammalian growth (FAO/WHO, 1973). The protein content in the HFD prevents severe decreases in carbohydrate intake and was significantly greater than the protein content used in low protein diet studies (Boujendar et al., 2002).

**FIGURE 1:**
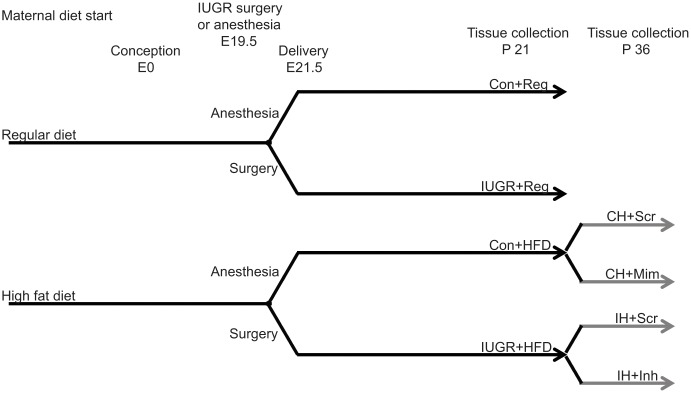
Schematic of study design. Rat dams were fed either a regular, standard rat chow or a high fat diet from 5 weeks prior to conception through the end of lactation. Pregnant dams underwent bilateral uterine artery ligation to produce IUGR offspring or anesthesia as a control. At P 21, offspring were either harvested after an overnight fast (black lines) or weaned from the dam to an HFD (gray lines). HFD fed IUGR and control offspring were injected with a microRNA-122 mimic, inhibitor, or scrambled sequence to test the function of miR-122 in hepatic cholesterol accumulation in this model.

After 5 weeks, males were placed in the female’s cage overnight for mating. Embryonic day 0.5 (E0.5) was determined either by presence of a plug, or in the absence of a plug, by the presence of sperm on a vaginal swab. To generate IUGR rats, bilateral uterine artery ligation was performed on E19.5 of a 21.5 day gestation, as previously described (Fu et al., 2006). Dams were allowed to deliver naturally and litters were culled to 6 at birth for rearing consistency. Our study design resulted in four offspring groups per sex: maternal regular diet-fed non-IUGR control rats (Con+Reg), maternal regular diet-fed IUGR rats (IUGR+Reg), maternal HFD-fed non-IUGR control rats (Con+HFD), and maternal HFD-fed IUGR rats (IUGR+HFD) (**Figure 1**).

At P 21, the standard time for weaning, rats were fasted overnight for 12 h prior to administration of 8 mg/kg xylazine and 40 mg/kg ketamine for anesthesia followed by decapitation. The left anterior lobe of the liver was flash-frozen and stored at -80°C until further analysis.

### Serum Lipid Analysis

Offspring blood was collected at the time of necropsy in serum separator tubes (BD Vacutainer, BD, Franklin Lakes, NJ). Blood was allowed to separate at room temperature for 30 min prior to centrifugal serum separation. Serum aliquots were frozen at -80°C immediately after separation. Serum lipid panels, aspartate aminotransferase (AST), alanine aminotransferase (ALT), and bicarbonate were analyzed at ARUP laboratories within 1 week of collection. Serum was analyzed from ten offspring per sex per group.

### Hepatic Cholesterol and Triglyceride Quantification

Hepatic lipids were isolated from frozen liver tissue based on the method developed by Folch et al. (1957) and as previously described (Zinkhan et al., 2014). Total hepatic cholesterol was measured using a colorimetric kit (BioVision HDL-C and LDL-C/VLDL-C Cholesterol Quantification Kit, Mountain View, CA, United States) according to manufacturer’s protocol. Hepatic triglyceride was measured using a colorimetric kit (BioVision Triglyceride Quantification Kit) according to manufacturer’s protocol. Data was calculated as mg cholesterol or mg triglyceride /100 g liver weight and compared to reference data. Lipids were isolated and cholesterol and triglycerides were analyzed from six offspring per sex per group.

### Hepatic Bile Acid Quantification

Hepatic bile acids were measured as a surrogate marker for bile acid levels in bile because rats do not have gall bladders. Hepatic bile acids were extracted from 100 mg frozen liver tissue crushed over liquid nitrogen as per a user supplied protocol from Crystal Chem, Inc. (Downers Grove, IL, United States). Crushed tissue was resuspended in 1 mL 75% ethanol for 2 h at 50°C. Twenty microliters of supernatant was used in a Rat Total Bile Acids kit (Crystal Chem, Inc.) per manufacturer’s instructions. Male and female bile acid samples were run on separate plates and thus not directly comparable. Bile acid samples were analyzed from six offspring per sex per group.

### Hepatic Protein Quantification

Hepatic protein was isolated using whole cell lysates in buffer (150 mM NaCl, 50 mM Tris pH 7.4, 1 mM EDTA, 0.25% Na-deoxycholate, 1% Igepal CA-630). Bicinchoninic acid assay, with bovine serum albumin as the standard, was used to measure protein concentrations. A total of 50 μg protein per sample was run on a 10% SDS–PAGE gel (Bio-Rad, Hercules, CA, United States). The protein was transferred to a PVDF membrane and blocked with 5% milk. Hypoxanthine phosphoribosyltransferase 1 (Hprt1) (15059-1-AP rabbit polyclonal antibody, Proteintech, Chicago, IL, United States) was used as a loading control, since Hprt1 did not differ by intrauterine environmental conditions or diet. Antibodies used in this study included Lxrα (LS-C172075, rabbit polyclonal antibody, LifeSpan BioSciences, Inc.), Cyp7a1 (sc-25536, RRID:AB_2088578, rabbit polyclonal antibody, Santa Cruz Biotechnology, Inc.), Srebp2 (ab28482, RRID:AB_778070, rabbit polyclonal antibody, Abcam, Cambridge, MA, United States), Hmgcr (NBP1-50713, rabbit polyclonal antibody, Novus Biologicals, Littleton, CO, United States), Ldlr (3839, RRID:AB_2281168, rabbit polyclonal antibody, BioVision), Abca1 (NB400-105, rabbit polyclonal antibody, Novus Biologicals), Abcg1 (sc-11150, RRID:AB_2220188, goat polyclonal antibody, SantaCruz Biotechnology), Fasn (610962, mouse monoclonal antibody, BD Transduction Laboratories, Santa Cruz, CA, United States), Mtp (612022, RRID:AB_399417, mouse monoclonal antibody, BD Transduction Laboratories). Western Lightning enhanced chemiluminescence (ECL) (PerkinElmer Life Sciences) was used to detect protein using either goat anti-rabbit or anti-mouse (for Fasn and Mtp) horseradish peroxidase conjugated secondary antibody (Cell Signaling Technology). A Kodak Image Station 2000ER (Eastman Kodak/SIS, Rochester, NY, United States) was used to visualize and quantify protein. Hepatic protein levels were analyzed from six offspring per sex per group.

### Hepatic RNA Isolation

Total hepatic RNA was isolated from the frozen left anterior lobe of liver as previously described using the RNeasy Lipid Tissue Mini Kit (Qiagen, Valencia, CA, United States) (Joss-Moore et al., 2010). A spectrophotometer was used to quantify total RNA. One μg hepatic RNA was used to synthesize cDNA using the High-Capacity cDNA Reverse Transcription Kit (Applied Biosystems, Foster City, CA) per manufacturers’ protocol.

### Hepatic mRNA Quantification

Semi-quantitative real-time reverse-transcriptase PCR was performed on hepatic cDNA using *Hprt1* message (Proteintech) as an internal control, since *C*_t_ values of hepatic *Hprt1* did not differ between intrauterine conditions. Relative quantification of target gene RT-PCR products was based on differences between *Hprt1* and the target gene using the comparative *C*_t_ method (TaqMan Gold RT-PCR manual; PE Biosystems, Foster City, CA, United States) (Livak and Schmittgen, 2001). *Cyp7a1, Adipocyte differentiation-related protein (Adrp), Carbohydrate responsive element binding protein (ChREBP), Peroxisome proliferator activated receptor alpha (Pparα), Pparγ, Sterol response element binding factor 1 (Srebf1), Srebf1c, Farnesoid X receptor (Fxr)*, and *Retinoid X receptor (Rxr)* messages were quantified using Taqman Gene Expression Assays (Applied Biosystems, Carlsbad, CA, United States).

### Hepatic miRNA Isolation

Total hepatic miRNA was isolated using mirVana miRNA Isolation Kit with phenol (Life Technologies, Ambion, Grand Island, NY, United States) per manufacturer’s protocol. Total miRNA was quantified using a spectrophotometer. The micro-cDNA was synthesized from 50 ng miRNA using the TaqMan MicroRNA Reverse Transcription Kit (Applied Biosystems) per manufacturer’s protocol, using miRNA specific primers for miR-122 and miR-103 as a control from TaqMan microRNA Assays (Applied Biosystems). Hepatic miRNA was isolated from six offspring per sex per group.

### Hepatic miRNA Quantification

Semi-quantitative Real-time RT PCR quantification was performed using miR-103 as an internal control, since *C*_t_ values of miR-103 did not differ between intrauterine conditions or after administration of miR-122 inhibitor, mimic, or scrambled sequence. Relative quantification of PCR products was based on differences between miR-103 and the target using the comparative *C*_t_ method (TaqMan Gold RT-PCR manual; PE Biosystems, Foster City, CA, United States) (Livak and Schmittgen, 2001). Assays were performed with TaqMan microRNA Assays (Applied Biosystems) for miR-122 and miR-103. Hepatic miRNA was analyzed from six offspring per sex per group.

### Hepatic Oil Red O Staining

A small piece of the left lobe of the liver was embedded in Optimal Cutting Temperature (VWR, Radnor, PA) and frozen. Embedded frozen liver was sliced to 6 μm thick slices, washed with 100% propylene glycol (ACROS Organics, Thermo Fisher Scientific, Fair Lawn, NJ, United States), stained with Oil Red O (Amresco, Solon, OH, United States), counterstained with Gill #2 modified haematoxylin, and mounted with Aquamount (Thermo Fisher Scientific). All slides were stained at the same time. Slides were visualized using a light microscope at 80× magnification. The bars in **Supplementary Figure S3** represent 50 μm.

### *In Vivo* miR-122 Experiments

The miR-122 mimic is a double-stranded oligonucleotide that mimics the function of endogenous miR-122 (Applied Biosystems). The miR-122 inhibitor is a single-stranded oligonucleotide designed to inhibit the endogenous miR-122 function (Applied Biosystems). A scrambled sequence of miR-122 was used as a negative control, designed with the same oligonucleotides as the miR-122 mimic but in a different order, and is thus unable to inhibit or mimic the function of miR-122 (Applied Biosystems). Con+HFD rats were injected with a miR-122 mimic to test the function of miR-122 in developing the IUGR+HFD phenotype or with a miR-122 scrambled sequence as a control. IUGR+HFD rats were injected with a miR-122 inhibitor to test the ability to normalize the IUGR+HFD phenotype or with a scrambled sequence as a control. Our study design resulted in 4 groups: Con+HFD rats injected with a scrambled sequence as a control (CH+Scr), Con+HFD rats injected with a miR-122 mimic (CH+Mim), IUGR+HFD rats injected with a scrambled sequence as a control (IH+Scr), and IUGR+HFD rats injected with a miR-122 inhibitor (IH+Inh).

At P 21, female rats were weaned to the same HFD as provided to the pregnant dams. MicroRNA-122 mimic, inhibitor, or scrambled sequence were administered via a single injection into a betadine and ethanol sterilized tail vein for a total dose of 2.5 mg/kg body weight. All rats had *ad libitum* access to HFD food and water through P 36. On P 36, the time of necropsy, rats were fasted overnight for 12 h followed by anesthesia administration of 8 mg/kg xylazine and 40 mg/kg ketamine for decapitation. Tissue collection and analysis was performed as described above.

### Statistics

Data tables and figures were expressed as scatter plots of individual rats with mean ± standard deviation (SD) shown. One-way ANOVA (with Fisher’s protected least-significant difference) was used for data analysis. A *p*-value of ≤ 0.05 was considered to be statistically significant. Male and female data were analyzed separately. Analysis was performed with GraphPad Prism version 7.01 (GraphPad Software, Inc., La Jolla, CA, United States).

## Results

### A Maternal HFD Increased Serum Cholesterol and Triglyceride Levels in IUGR and Control Rats

**FIGURE 2:**
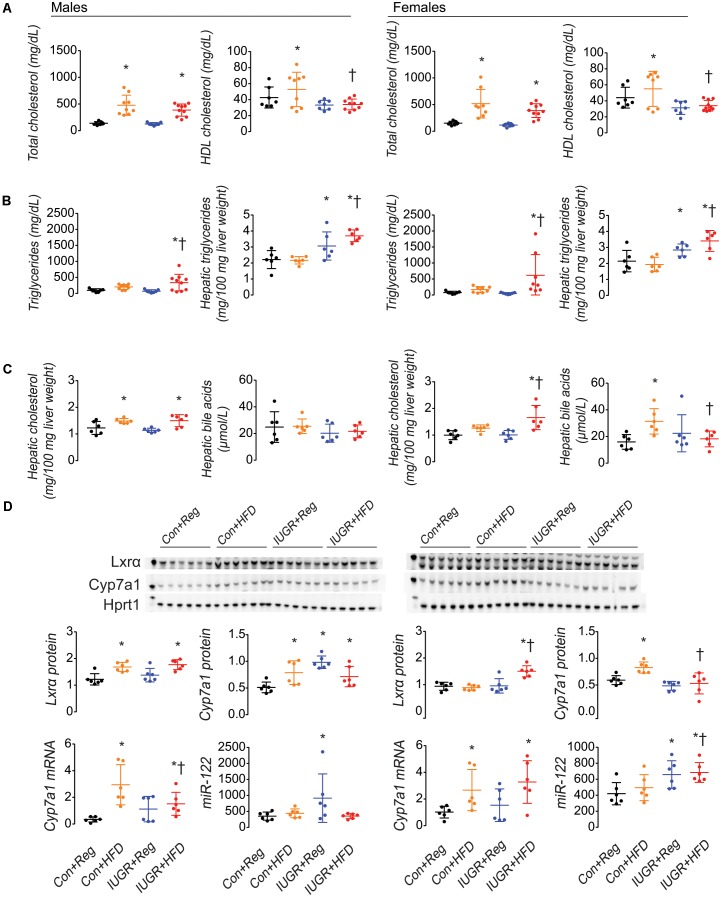
IUGR+HFD female rats had decreased serum HDL **(A)** compared to Con+HFD female rats, increased hepatic triglycerides **(B)** and cholesterol **(C)**, decreased hepatic bile acids **(C)**, decreased Cyp7a1 protein, and increased miR-122 **(D)** compared to Con+Reg female rats. A maternal HFD increased cholesterol and hepatic Cyp7a1 mRNA and protein in both sexes in IUGR and control rats **(D)**. Data shown as scatter plots of individual rats with mean ± SD with a minimum *n* = 6 rats per sex, per intrauterine environment, each from separate litters. Data from male rats is shown on the left of the figure, and from female rats is shown on the right. Groups are denoted as follows: Con+Reg data are shown in black, Con+HFD data are shown in yellow, IUGR+Reg data are shown in blue, and IUGR+HFD data are shown in red, with group names listed below the graphs on the bottom of the figure. Western blot images of Lxrα, Cyp7a1, and Hprt1 protein are shown above the graphical representation of band densitometry. The kilodalton (kDa) marker is shown on the left lane of the western blot image; the 50 kDa marker is shown in the Lxrα blot, the 60 kDa marker is shown in the Cyp7a1 blot, and the 30 kDa marker is shown in the Hprt1 blot and the Hprt1 image was obtained from the blot with Cyp7a1. A *p*-value ≤0.05 is denoted with an asterisk (^∗^) for any group data compared to sex-matched Con+Reg, and a *p*-value ≤ 0.05 is denoted with a hatched line (†) for IUGR+HFD data compared to sex-matched Con+HFD.

Serum total cholesterol was increased in both Con+HFD and IUGR+HFD male and female rats compared to sex-matched Con+Reg rats (**Figure 2A**). Serum HDL cholesterol was increased in Con+HFD rats compared to sex-matched Con+Reg rats, but IUGR+HFD rats had decreased HDL cholesterol compared to Con+HFD rats. Compared to sex-matched Con+Reg rats, IUGR+Reg rats did not have changes in serum cholesterol levels measured in this study. Serum triglycerides were increased in male and female IUGR+HFD rats compared to sex-matched Con+Reg rats and Con+HFD rats (**Figure 2B**). Maternal HFD feeding and IUGR qualitatively increased hepatic oil red o stain intensity in Con+HFD and IUGR+HFD male and female rats (**Supplementary Figure S3**).

### IUGR Combined With a Maternal HFD Increased Hepatic Cholesterol and Triglycerides in Female Rats

Female IUGR+HFD rats had increased hepatic cholesterol compared to both Con+Reg and Con+HFD female rats (**Figure 2C**). Male Con+HFD and IUGR+HFD rats had increased hepatic cholesterol compared to Con+Reg male rats, with no difference between IUGR+HFD and Con+HFD hepatic cholesterol levels. IUGR+Reg rats did not have increased hepatic cholesterol compared to Con+Reg rats. Male and female IUGR+HFD rats had increased hepatic triglyceride compared to sex-matched Con+Reg and Con+HFD rats (**Figure 2B**). IUGR+Reg male and female rats had increased hepatic triglyceride compared to Con+Reg rats.

### IUGR Combined With a Maternal HFD Increased Hepatic Bile Acids in Female Rats

Female Con+HFD rats had increased hepatic bile acids compared to Con+Reg rats, and IUGR+HFD rats had decreased hepatic bile acids compared to Con+HFD rats (**Figure 2C**). There was no difference in hepatic bile acids between any of the male rat groups.

### IUGR Combined With a Maternal HFD Decreased Hepatic Cyp7a1 Protein in Female Rats

Despite increased hepatic cholesterol accumulation in IUGR+HFD female rats, IUGR+HFD female rats had decreased protein levels of Cyp7a1 (**Figure 2D**). This was surprising because IUGR+HFD female rats had increased levels of Lxrα protein (**Figure 2D**). IUGR+HFD female rats also had decreased Abca1, Abcg1 (**Supplementary Figure S1A**), Fasn, and Mtp protein levels (**Supplementary Figure S1B**), and had increased protein levels of Srebp2 compared to Con+HFD female rats (**Supplementary Figure S1C**). There was no difference between IUGR+HFD and Con+HFD female rats for hepatic protein levels of Hmgcr and Ldlr (**Supplementary Figure S1C**). Without increased hepatic cholesterol levels, Con+HFD female rats had increased hepatic protein levels of Cyp7a1 (**Figure 2D**), Abca1, Abcg1 (**Supplementary Figure S1A**), and Fasn (**Supplementary Figure S1B**) compared to Con+Reg female rats.

Male rats, male Con+HFD and IUGR+HFD rats had increased protein levels of Lxrα, Cyp7a1 (**Figure 2D**), Abca1, Abcg1 (**Supplementary Figure S1A**), and Ldlr (**Supplementary Figure S1C**), decreased protein levels of Fasn and Mtp (**Supplementary Figure S1B**). IUGR+HFD male rats had further increased hepatic protein levels of Abca1 (**Supplementary Figure S1A**) and Srebp2 (**Supplementary Figure 1C**) compared to both Con+HFD and Con+Reg male rats.

### IUGR Combined With a Maternal HFD Increased Hepatic Cyp7a1 Messenger RNA and MicroRNA-122 in Female Rats

*Cyp7a1* mRNA levels were increased in Con+HFD and IUGR+HFD male and female rats compared to sex-matched Con+Reg rats (**Figure 2D**). IUGR+Reg rats did not have increased *Cyp7a1* mRNA levels.

IUGR+HFD female but not male rats had increased miR-122 levels compared to Con+HFD rats (**Figure 2D**). IUGR+Reg male and female rats had increased miR-122 levels compared to Con+Reg rats.

Lipogenic genes *Adrp* and *ChREBP* were increased in IUGR+Reg, Con+HFD, and IUGR+HFD female rats compared to Con+Reg rats (**Supplementary Figure S2**). *Adrp* was increased in male IUGR+Reg and IUGR+HFD rats compared to Con+Reg and Con+HFD rats. There was no difference between groups for *Pparα, Pparγ, Srebf1*, *Srebf1c, Fxr*, or *Rxr* mRNA levels.

### Inhibition of MicroRNA-122 Increased Cyp7a1 Protein and Decreased Hepatic Cholesterol in IUGR Rats Exposed to a Maternal HFD

**FIGURE 3:**
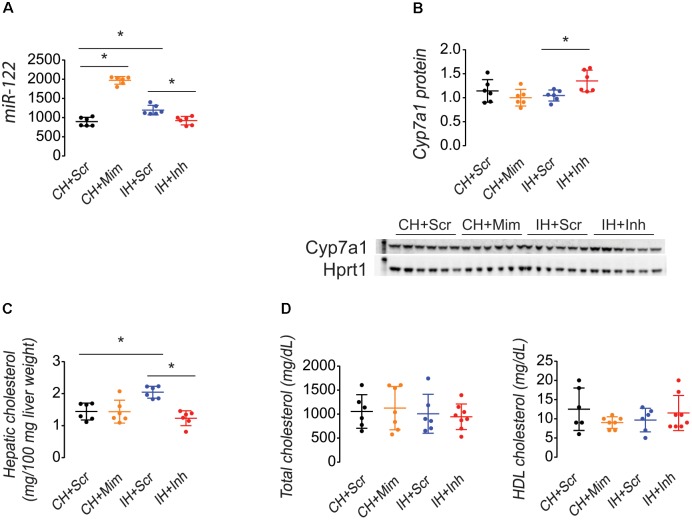
Injection of a miR-122 inhibitor into IUGR+HFD female rats decreased hepatic miR-122 **(A)**, increased Cyp7a1 protein **(B)**, and decreased hepatic cholesterol **(C)**. Injections did not change serum total or HDL cholesterol **(D)**. Data shown as scatter plots of individual rats with mean ± SD with a minimum *n* = 6 rats per sex, per intrauterine environment, per type of injection, each from separate litters. Groups are denoted as follows: CH+Scr data are shown in black, CH+Mim data are shown in yellow, IH+Scr data are shown in blue, and IH+Inh data are shown in red. Western blot images of Cyp7a1 and Hprt1 protein are shown above the graphical representation of band densitometry. The kilodalton (kDa) marker is shown on the left lane of the western blot image; the 60 kDa marker is shown in the Cyp7a1 blot, and the 30 kDa marker is shown in the Hprt1 blot. A *p*-value ≤ 0.05 is denoted with an asterisk (^∗^) for any group data that differed as noted by the line above the graph.

Compared to injection of miR-122 scrambled sequence as a control, injection of a miR-122 mimic to Con+HFD female rats increased hepatic miR-122 levels and administration of a miR-122 inhibitor to IUGR+HFD female rats decreased miR-122 levels (**Figure 3A**). Injection of a miR-122 inhibitor increased hepatic Cyp7a1 protein levels (**Figure 3B**) and decreased hepatic cholesterol levels (**Figure 3C**) in IUGR+HFD female rats. Injection of a miR-122 mimic to Con+HFD female rats did not increase hepatic cholesterol or decrease hepatic Cyp7a1 protein. None of the injections altered serum total or HDL cholesterol compared to injection of the miR-122 scrambled sequence (**Figure 3D**).

## Discussion

The primary finding in this study is that female rats subjected to IUGR and a maternal HFD had increased hepatic cholesterol, decreased hepatic Cyp7a1 protein and bile acids, and increased miR-122. Inhibition of miR-122 in IUGR+HFD female rats increased Cyp7a1 protein and decreased hepatic cholesterol in IUGR+HFD rats. These findings suggest that one pathway through which IUGR induces hepatic cholesterol accumulation may be via increased miR-122 inhibition of Cyp7a1, causing decreased cholesterol catabolism to bile acids.

Cholesterol catabolism to bile acids via Cyp7a1 eliminates approximately 50–70% of cholesterol undergoing elimination (Vlahcevic et al., 1999). The most highly regulated and rate limiting step in cholesterol catabolism to bile acids is regulation of Cyp7a1. Cyp7a1 overexpression in cell culture increases bile acid synthesis and decreases cholesterol levels (Spady et al., 1995; Pandak et al., 2001). Increasing Cyp7a1 expression has been postulated as a potential treatment for hypercholesterolemia. Deficiency in Cyp7a1 in mice leads to high serum and hepatic cholesterol levels and decreased bile acid formation (Erickson et al., 2003). Interestingly, in Cyp7a1 knockout mice, decreased Cyp7a1 was not accompanied by increases in the minor bile acid metabolizing enzymes (Schwarz et al., 2001). The perinatal environment also influences Cyp7a1 expression. Protein-restriction induced IUGR rats have decreased Cyp7a1 and increased cholesterol in adult males (Sohi et al., 2011a). Similarly, our previous study demonstrated decreased Cyp7a1 protein and increased serum and hepatic cholesterol in adult IUGR rats weaned to a HFD (Zinkhan et al., 2014). Together, these findings suggest that cholesterol catabolism to bile acids via Cyp7a1 is susceptible to the in utero nutritional environment. The susceptibility of Cyp7a1 expression to the perinatal environment can result in persistent decreases in Cyp7a1 and increases in cholesterol accumulation in the liver.

Alteration of Cyp7a1 levels may be via several mechanisms. First, transcriptional regulation of Cyp7a1 occurs via transcription factors such as Lxrα. Lxrα responds to lipid overload by binding to lipids, leading to increased transcription of effector genes such as Cyp7a1 and promoting cholesterol excretion in bile acids (Ide et al., 2003; Yoshikawa et al., 2003). In this study, both Lxrα protein and *Cyp7a1* mRNA were increased in IUGR+HFD female rat livers, but not Cyp7a1 protein levels. The difference between *Cyp7a1* mRNA and protein levels suggests that the decreased Cyp7a1 protein occurred via a different pathway than transcriptional regulation. Regulation of Cyp7a1 also occurs via post-transcriptional regulation by microRNAs such as miR-122 (Song et al., 2010). MicroRNA-122 regulates Cyp7a1 via inhibition of mRNA translation, and may explain the difference between increased mRNA and decreased protein levels of Cyp7a1 in IUGR+HFD female rats (Song et al., 2010). Inhibition of miR-122 in IUGR+HFD rats partially increased Cyp7a1 protein and decreased hepatic cholesterol levels, suggesting that miR-122 plays a role in hepatic cholesterol accumulation in our rat model. However, we were unable to replicate IUGR+HFD rat cholesterol pathology with administration of a miR-122 mimic, suggesting the role of other pathways in inducing hepatic cholesterol accumulation in IUGR+HFD rats. These other pathways may include regulation of Cyp7a1 via other microRNAs such as miR-422, extra-hepatic regulation of Cyp7a1 through intestinal Fibroblast growth factor 15, reduction of Cyp7a1 mRNA stability by bile acids, or alteration of other enzymes involved in hepatic cholesterol accumulation (Agellon and Cheema, 1997; Song et al., 2010; Out et al., 2011). Unlike microRNA inhibitors, microRNA mimics cannot be extensively modified to enhance stability and function (as reviewed in Thakral and Ghoshal, 2015). While a 2.5 mg/kg dose of a miR-122 inhibitor was able to normalize Cyp7a1 protein, a larger dose of the miR-122 mimic may have been needed to replicate IUGR+HFD pathophysiology in our rat model.

High density lipoprotein cholesterol returns cholesterol from the blood and vasculature to the liver through reverse cholesterol transport and delivers cholesterol to enterocytes for direct elimination. The liver regulates HDL cholesterol levels in part via Abca1 and Abcg1, proteins that allow for HDL export from the liver into circulation. Little is known regarding the susceptibility of Abca1 and Abcg1 to the perinatal environment. In humans, fetal IUGR and maternal preeclampsia decrease Abca1 and Abcg1 expression (Baumann et al., 2013). In our study, IUGR+HFD female rats had decreased Abca1 and Abcg1 protein abundance and decreased HDL cholesterol. Decreased Abca1 and Abcg1 protein abundance may suggest that expression of these proteins is susceptible to the perinatal environment.

Intrauterine growth restriction induces sex-specific increases in cholesterol levels in humans and rodents (Forsdahl, 1978; Sohi et al., 2011b). In humans, IUGR men have an increased LDL-C to HDL-C ratio compared to IUGR women (Robinson et al., 2006). In rodents, dietary supplementation with cholesterol induces sex-specific responses in Cyp7a1 expression. *Cyp7a1* knock-out female mice demonstrate increased cholesterol absorption and cholesterol levels, while *Cyp7a1* knock-out male mice demonstrate minimal increase in cholesterol absorption and cholesterol levels (Schwarz et al., 2001). When fed a healthy diet, *Ldlr* knock-out female mice had higher cholesterol compared to male *Ldlr* knock-out mice (Hatch et al., 2012). In our previous study female IUGR rats fed a HFD in adulthood had higher serum cholesterol levels than male IUGR rats fed the same diet even when IUGR decreased Cyp7a1 protein in both sexes (Zinkhan et al., 2014). Sex steroids may impact regulation of cholesterol levels. Ovariectomy did not increase cholesterol in females, but orchidectomy increased cholesterol in males (Hatch et al., 2012). Cumulatively, these findings suggest that androgen levels may protect against hypercholesterolemia, and consumption of a HFD may interact with sex steroids to impact cholesterol metabolism (Hatch et al., 2012).

Intrauterine growth restriction also induced an increase in serum and hepatic triglycerides and hepatic oil red o staining in our rat model, particularly when combined with a high fat diet. Human studies indicate a correlation between birthweight and serum triglyceride levels in adulthood. The highest triglycerides in adulthood were found in those born at the lowest birthweight both in the Hertfordshire cohort consisting of 297 women (Fall et al., 1995) and a separate Hertfordshire cohort consisting of 370 men (Phillips et al., 1998). Many studies have also shown increased triglycerides in a variety of IUGR animal models. IUGR female baboons have increased LDL cholesterol and a trend for increased triglycerides in adulthood (Kuo et al., 2018). Male and female IUGR Yucatan miniature pigs had increase serum and hepatic triglycerides in adulthood (Myrie et al., 2017). IUGR rats had increased serum and hepatic triglycerides in early adulthood (Chen et al., 2016). In our study, increased serum and hepatic triglycerides were associated with increased hepatic expression of *Adrp* mRNA in male and female IUGR+Reg and IUGR+HFD rats and *ChREBP* mRNA in female IUGR+Reg and IUGR+HFD rats. Adrp and ChREBP have been shown to be affected by the intrauterine environment. Bone marrow mesenchymal stem cells from IUGR rats showed an increased adipogenic profile including increased *Adrp* mRNA and protein (Gong et al., 2016). Increased hepatic lipids were associated with increased ChREBP in a mouse model of maternal magnesium deficiency (Gupta et al., 2014). These findings suggest that genes involved in lipid metabolism are sensitive to the intrauterine environment and may play a role in the increased risk for high triglycerides later in life.

A limitation of this study is that unlike in humans, the HFD fed rats in this study consumed the same total kilocalories per day as regular diet fed rats. Self-regulation of food intake benefited interpretation of the results of this study as caloric intake was similar between the HFD fed rats and regular diet fed rats, and thus unlikely to have a significant impact on the difference between the regular diet and HFD fed rats. Further, the protein content of the HFD is lower than the regular diet. The protein content in the HFD was chosen to mimic protein consumption in the United States today (Wright et al., 2003) and is sufficient for normal mammalian growth (FAO/WHO, 1973). This protein content is significantly higher than protein content used in low protein dietary studies (Boujendar et al., 2002). Lastly, as with all animal studies, caution should be taken with correlating findings from this study to the human condition.

In conclusion, female IUGR rats exposed to a maternal HFD had increased hepatic cholesterol accumulation, decreased hepatic Cyp7a1 protein, decreased hepatic bile acids, and increased hepatic miR-122. Inhibition of miR-122 decreased hepatic cholesterol and increased Cyp7a1 protein levels in IUGR+HFD female rats, suggesting that IUGR and maternal HFD-induced hepatic cholesterol accumulation occurs in part through increased hepatic miR-122.

## Data Availability Statement

The raw data supporting the conclusions of this manuscript will be made available by the authors, without undue reservation, to any qualified researcher.

## Author Contributions

EZ and AS designed the study and wrote the manuscript. EZ and BY performed the experiments. EZ performed the statistical analysis. All authors discussed the results and commented on the manuscript.

## Conflict of Interest Statement

The authors declare that the research was conducted in the absence of any commercial or financial relationships that could be construed as a potential conflict of interest.

## Supplementary Material

FIGURE S1 IUGR+HFD female rats had decreased Abca1, Abcg1 **(A)**, Fasn, and Mtp protein **(B)**, and did not change Srebp2, Ldlr, or Hmgcr protein **(C)**. Data shown as scatter plots of individual rats with mean ± SD for *n* = 6 rats per sex, per intrauterine environment, each from separate litters. Data from male rats is shown on the left of the figure, and from female rats is shown on the right. Groups are denoted as follows: Con+Reg data are shown in black, Con+HFD data are shown in yellow, IUGR+Reg data are shown in blue, and IUGR+HFD data are shown in red, with group names listed below the graphs on the bottom of the figure. Western blot images are shown above the graphical representation of band densitometry. The kilodalton (kDa) marker is shown on the left lane of the western blot image; the 220 kDa marker is shown in the Abca1 blot, the 80 and 100 kDa markers are shown in the Abcg1 blot, the 220 kDa marker is shown in the Fasn blot, the 80 and 100 kDa markers are shown in the Mtp blot, the 50 and 60 kDa markers are shown in the Srebp2 blot with no precursor band visualized for Srebp2 protein, The 80 and 100 kDa markers are shown in the Ldlr blot, the 80 and 100 kDa markers are shown in the top Hmgcr blot and 50 kDa band is shown in the bottom Hmgcr blot, and the 30 kDa marker is shown in the Hprt1 blot and the Hprt1 image was obtained from the blot with Hmgcr. A *p*-value ≤ 0.05 is denoted with an asterisk (^∗^) for any group data compared to sex-matched Con+Reg, and a *p*-value ≤ 0.05 is denoted with a hatched line (†) for IUGR+HFD data compared to sex-matched Con+HFD.

FIGURE S2 IUGR+Reg and IUGR+HFD male and female rats had increased *Adrp* mRNA, and IUGR+Reg and IUGR+HFD female rats had increased *ChREBP* mRNA. There were no differences in *Pparα, Pparγ, Srebf1, Srebf1c, Fxr*, or *Rxr* mRNA levels. Data shown as scatter plots of individual rats with mean ± SD for *n* = 6 rats per sex, per intrauterine environment, each from separate litters. Data from male rats is shown on the left of the figure, and from female rats is shown on the right. Groups are denoted as follows: Con+Reg data are shown in black, Con+HFD data are shown in yellow, IUGR+Reg data are shown in blue, and IUGR+HFD data are shown in red, with group names listed below the graphs on the bottom of the figure.

FIGURE S3 Images of left lobe of the liver stained with Oil Red O and hematoxylin. Bar represents 50 μm.

## References

[B1] Agellon L. B. Cheema S. K. (1997). The 3’-untranslated region of the mouse cholesterol 7alpha-hydroxylase mRNA contains elements responsive to post-transcriptional regulation by bile acids. *Biochem. J.* 328(Pt 2) 393–399. 10.1042/bj3280393 9371693 PMC1218933

[B2] Barker D. J. Martyn C. N. Osmond C. Hales C. N. Fall C. H. (1993). Growth in utero and serum cholesterol concentrations in adult life. *BMJ* 307 1524–1527. 10.1136/bmj.307.6918.1524 8274921 PMC1679540

[B3] Barker D. J. Winter P. D. Osmond C. Margetts B. Simmonds S. J. (1989). Weight in infancy and death from ischaemic heart disease. *Lancet* 2 577–580. 10.1016/S0140-6736(89)90710-1 2570282

[B4] Baumann M. Korner M. Huang X. Wenger F. Surbek D. Albrecht C. (2013). Placental ABCA1 and ABCG1 expression in gestational disease: pre-eclampsia affects ABCA1 levels in syncytiotrophoblasts. *Placenta* 34 1079–1086. 10.1016/j.placenta.2013.06.309 23880356

[B5] Boujendar S. Reusens B. Merezak S. Ahn M. T. Arany E. Hill D. (2002). Taurine supplementation to a low protein diet during foetal and early postnatal life restores a normal proliferation and apoptosis of rat pancreatic islets. *Diabetologia* 45 856–866. 10.1007/s00125-002-0833-6 12107730

[B6] Chen L. H. Liang L. Fang Y. L. Wang Y. M. Zhu W. F. (2016). Fish oil improves lipid profile in juvenile rats with intrauterine growth retardation by altering the transcriptional expression of lipid-related hepatic genes. *J. Matern. Fetal Neonatal Med.* 29 3292–3298. 10.3109/14767058.2015.1123244 26586306

[B7] Erickson S. K. Lear S. R. Deane S. Dubrac S. Huling S. L. Nguyen L. (2003). Hypercholesterolemia and changes in lipid and bile acid metabolism in male and female cyp7A1-deficient mice. *J. Lipid Res.* 44 1001–1009. 10.1194/jlr.M200489-JLR200 12588950

[B8] Fall C. H. Osmond C. Barker D. J. Clark P. M. Hales C. N. Stirling Y. (1995). Fetal and infant growth and cardiovascular risk factors in women. *BMJ* 310 428–432. 10.1136/bmj.310.6977.428 7873947 PMC2548816

[B9] FAO/WHO (1973). Energy and protein requirements. Report of a joint FAO/WHO ad hoc expert committee. *Nutr. Meet Rep. Ser.* 52 1–118. 4220351

[B10] Folch J. Lees M. Sloane Stanley G. H. (1957). A simple method for the isolation and purification of total lipides from animal tissues. *J. Biol. Chem.* 226 497–509. 13428781

[B11] Forsdahl A. (1978). Living conditions in childhood and subsequent development of risk factors for arteriosclerotic heart disease. The cardiovascular survey in Finnmark 1974-75. *J. Epidemiol. Commun. Health* 32 34–37. 10.1136/jech.32.1.34 262586 PMC1087307

[B12] Fu Q. McKnight R. A. Yu X. Callaway C. W. Lane R. H. (2006). Growth retardation alters the epigenetic characteristics of hepatic dual specificity phosphatase 5. *FASEB J.* 20 2127–2129. 10.1096/fj.06-6179fje 16940436

[B13] Gong M. Antony S. Sakurai R. Liu J. Iacovino M. Rehan V. K. (2016). Bone marrow mesenchymal stem cells of the intrauterine growth-restricted rat offspring exhibit enhanced adipogenic phenotype. *Int. J. Obes.* 40 1768–1775. 10.1038/ijo.2016.157 27599633 PMC5113998

[B14] Gupta M. Solanki M. H. Chatterjee P. K. Xue X. Roman A. Desai N. (2014). Maternal magnesium deficiency in mice leads to maternal metabolic dysfunction and altered lipid metabolism with fetal growth restriction. *Mol. Med.* 20 332–340. 10.2119/molmed.2014.00137 25025397 PMC4153836

[B15] Hatch N. W. Srodulski S. J. Chan H. W. Zhang X. Tannock L. R. King V. L. (2012). Endogenous androgen deficiency enhances diet-induced hypercholesterolemia and atherosclerosis in low-density lipoprotein receptor-deficient mice. *Gender Med.* 9 319–328. 10.1016/j.genm.2012.08.003 22981166 PMC3483072

[B16] Ide T. Shimano H. Yoshikawa T. Yahagi N. Amemiya-Kudo M. Matsuzaka T. (2003). Cross-talk between peroxisome proliferator-activated receptor (PPAR) alpha and liver X receptor (LXR) in nutritional regulation of fatty acid metabolism. II. LXRs suppress lipid degradation gene promoters through inhibition of PPAR signaling. *Mol. Endocrinol.* 17 1255–1267. 10.1210/me.2002-0191 12730332

[B17] Joss-Moore L. A. Wang Y. Campbell M. S. Moore B. Yu X. Callaway C. W. (2010). Uteroplacental insufficiency increases visceral adiposity and visceral adipose PPARgamma2 expression in male rat offspring prior to the onset of obesity. *Early Hum. Dev.* 86 179–185. 10.1016/j.earlhumdev.2010.02.006 20227202 PMC2857740

[B18] Kuo A. H. Li C. Mattern V. Huber H. F. Comuzzie A. Cox L. (2018). Sex-dimorphic acceleration of pericardial, subcutaneous, and plasma lipid increase in offspring of poorly nourished baboons. *Int. J. Obes.* 10.1038/s41366-018-0008-2 [Epub ahead of print]. 29463919 PMC6019612

[B19] Livak K. J. Schmittgen T. D. (2001). Analysis of relative gene expression data using real-time quantitative PCR and the 2(-Delta Delta C(T)) Method. *Methods* 25 402–408. 10.1006/meth.2001.1262 11846609

[B20] Myrie S. B. McKnight L. L. King J. C. McGuire J. J. Van Vliet B. N. Cheema S. K. (2017). Intrauterine growth-restricted Yucatan miniature pigs experience early catch-up growth, leading to greater adiposity and impaired lipid metabolism as young adults. *Appl. Physiol. Nutr. Metab.* 42 1322–1329. 10.1139/apnm-2017-0311 28813611

[B21] Out C. Hageman J. Bloks V. W. Gerrits H. Sollewijn Gelpke M. D. Bos T. (2011). Liver receptor homolog-1 is critical for adequate up-regulation of Cyp7a1 gene transcription and bile salt synthesis during bile salt sequestration. *Hepatology* 53 2075–2085. 10.1002/hep.24286 21391220

[B22] Pandak W. M. Schwarz C. Hylemon P. B. Mallonee D. Valerie K. Heuman D. M. (2001). Effects of CYP7A1 overexpression on cholesterol and bile acid homeostasis. *Am. J. Physiol. Gastrointest. Liver Physiol.* 281 G878–G889. 10.1152/ajpgi.2001.281.4.G878 11557507

[B23] Phillips D. I. Barker D. J. Fall C. H. Seckl J. R. Whorwood C. B. Wood P. J. (1998). Elevated plasma cortisol concentrations: a link between low birth weight and the insulin resistance syndrome? *J. Clin. Endocrinol. Metab.* 83 757–760. 10.1210/jcem.83.3.4634 9506721

[B24] Reynolds R. M. Allan K. M. Raja E. A. Bhattacharya S. McNeill G. Hannaford P. C. (2013). Maternal obesity during pregnancy and premature mortality from cardiovascular event in adult offspring: follow-up of 1 323 275 person years. *BMJ* 347:f4539. 10.1136/bmj.f4539 23943697 PMC3805484

[B25] Robinson S. M. Batelaan S. F. Syddall H. E. Sayer A. A. Dennison E. M. Martin H. J. (2006). Combined effects of dietary fat and birth weight on serum cholesterol concentrations: the Hertfordshire Cohort Study. *Am. J. Clin. Nutr.* 84 237–244. 10.1093/ajcn/84.1.237 16825701

[B26] Schwarz M. Russell D. W. Dietschy J. M. Turley S. D. (2001). Alternate pathways of bile acid synthesis in the cholesterol 7alpha-hydroxylase knockout mouse are not upregulated by either cholesterol or cholestyramine feeding. *J. Lipid Res.* 42 1594–1603. 11590215

[B27] Sohi G. Marchand K. Revesz A. Arany E. Hardy D. B. (2011a). Maternal protein restriction elevates cholesterol in adult rat offspring due to repressive changes in histone modifications at the cholesterol 7alpha-hydroxylase promoter. *Mol. Endocrinol.* 25 785–798. 10.1210/me.2010-0395 21372147 PMC5417260

[B28] Sohi G. Revesz A. Hardy D. B. (2011b). Permanent implications of intrauterine growth restriction on cholesterol homeostasis. *Sem. Reprod. Med.* 29 246–256. 10.1055/s-0031-1275523 21710400

[B29] Song K. H. Li T. Owsley E. Chiang J. Y. (2010). A putative role of micro RNA in regulation of cholesterol 7alpha-hydroxylase expression in human hepatocytes. *J. Lipid Res.* 51 2223–2233. 10.1194/jlr.M004531 20351063 PMC2903801

[B30] Spady D. K. Cuthbert J. A. Willard M. N. Meidell R. S. (1995). Adenovirus-mediated transfer of a gene encoding cholesterol 7 alpha-hydroxylase into hamsters increases hepatic enzyme activity and reduces plasma total and low density lipoprotein cholesterol. *J. Clin. Invest.* 96 700–709. 10.1172/JCI118113 7635963 PMC185253

[B31] Thakral S. Ghoshal K. (2015). miR-122 is a unique molecule with great potential in diagnosis, prognosis of liver disease, and therapy both as miRNA mimic and antimir. *Curr. Gene Ther.* 15 142–150. 10.2174/1566523214666141224095610 25537773 PMC4439190

[B32] Vlahcevic Z. R. Pandak W. M. Stravitz R. T. (1999). Regulation of bile acid biosynthesis. *Gastroenterol. Clin. North Am.* 28 1–25. 10.1016/S0889-8553(05)70041-8 10198776

[B33] Wright J. D. Wang C. Y. Kennedy-Stephenson J. Ervin R. B. (2003). Dietary intake of ten key nutrients for public health, United States: 1999-2000. *Adv. Data* 334 1–4. 12743879

[B34] Yoshikawa T. Ide T. Shimano H. Yahagi N. Amemiya-Kudo M. Matsuzaka T. (2003). Cross-talk between peroxisome proliferator-activated receptor (PPAR) alpha and liver X receptor (LXR) in nutritional regulation of fatty acid metabolism. I. PPARs suppress sterol regulatory element binding protein-1c promoter through inhibition of LXR signaling. *Mol. Endocrinol.* 17 1240–1254. 10.1210/me.2002-0190 12730331

[B35] Zinkhan E. K. Chin J. R. Zalla J. M. Yu B. Numpang B. Yu X. (2014). Combination of intrauterine growth restriction and a high-fat diet impairs cholesterol elimination in rats. *Pediatr. Res.* 76 432–440. 10.1038/pr.2014.117 25119340

