# Supplementary figures and images for: Prenatal Exposure to a Maternal High Fat Diet Increases Hepatic Cholesterol Accumulation in Intrauterine Growth Restricted Rats in Part Through MicroRNA-122 Inhibition of Cyp7a1
Source: Front Physiol. 2018 May 29;9:645. doi: 10.3389/fphys.2018.00645 (PMC5987111; doi:10.3389/fphys.2018.00645)

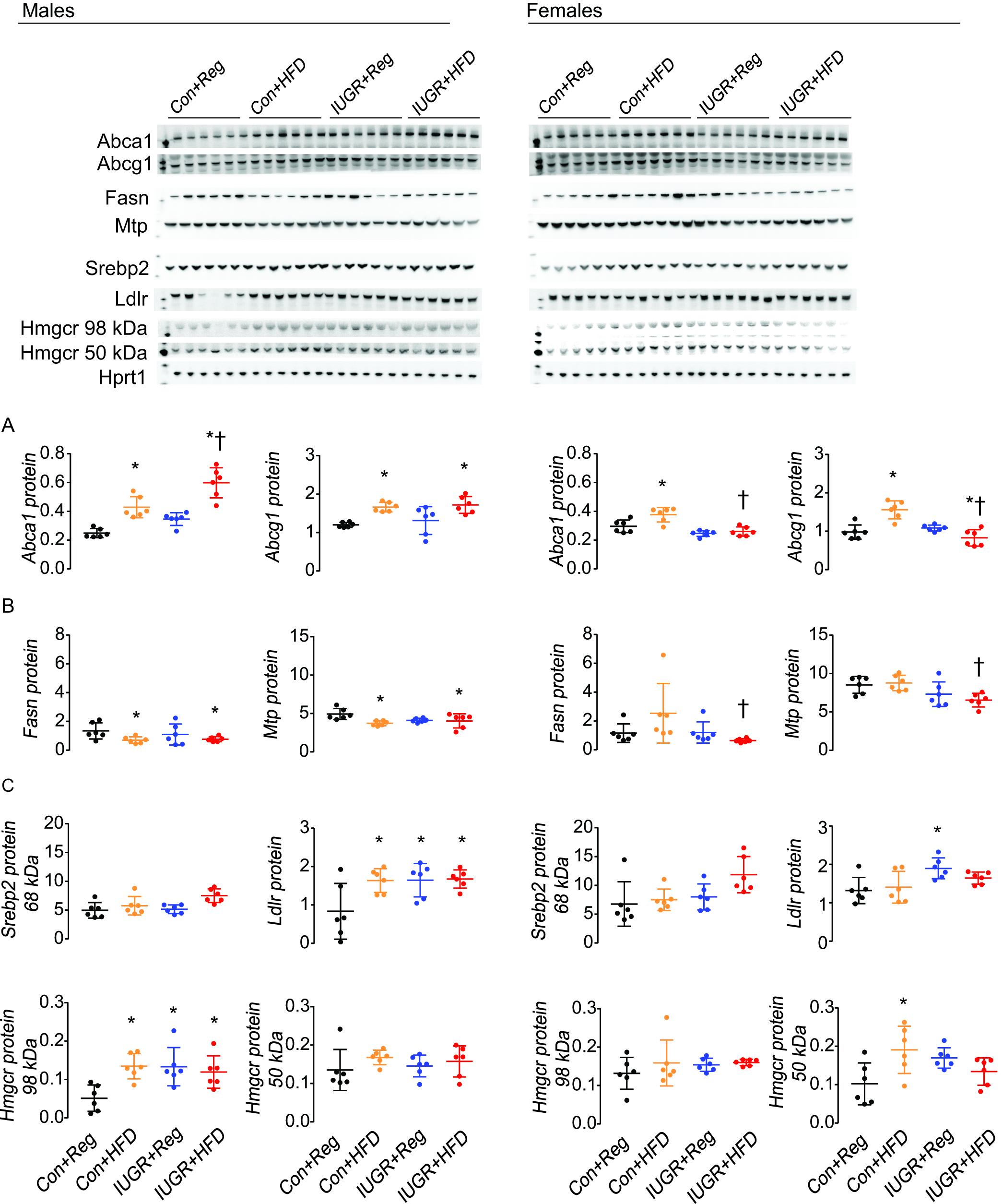

Supplement: FIGURE S1 — IUGR+HFD female rats had decreased Abca1, Abcg1 (A), Fasn, and Mtp protein (B), and did not change Srebp2, Ldlr, or Hmgcr protein (C). Data shown as scatter plots of individual rats with mean ± SD for n = 6 rats per sex, per intrauterine environment, each from separate litters. Data from male rats is shown on the left of the figure, and from female rats is shown on the right. Groups are denoted as follows: Con+Reg data are shown in black, Con+HFD data are shown in yellow, IUGR+Reg data are shown in blue, and IUGR+HFD data are shown in red, with group names listed below the graphs on the bottom of the figure. Western blot images are shown above the graphical representation of band densitometry. The kilodalton (kDa) marker is shown on the left lane of the western blot image; the 220 kDa marker is shown in the Abca1 blot, the 80 and 100 kDa markers are shown in the Abcg1 blot, the 220 kDa marker is shown in the Fasn blot, the 80 and 100 kDa markers are shown in the Mtp blot, the 50 and 60 kDa markers are shown in the Srebp2 blot with no precursor band visualized for Srebp2 protein, The 80 and 100 kDa markers are shown in the Ldlr blot, the 80 and 100 kDa markers are shown in the top Hmgcr blot and 50 kDa band is shown in the bottom Hmgcr blot, and the 30 kDa marker is shown in the Hprt1 blot and the Hprt1 image was obtained from the blot with Hmgcr. A p-value ≤ 0.05 is denoted with an asterisk (∗) for any group data compared to sex-matched Con+Reg, and a p-value ≤ 0.05 is denoted with a hatched line (†) for IUGR+HFD data compared to sex-matched Con+HFD. [file Image_1.TIF]

# Males

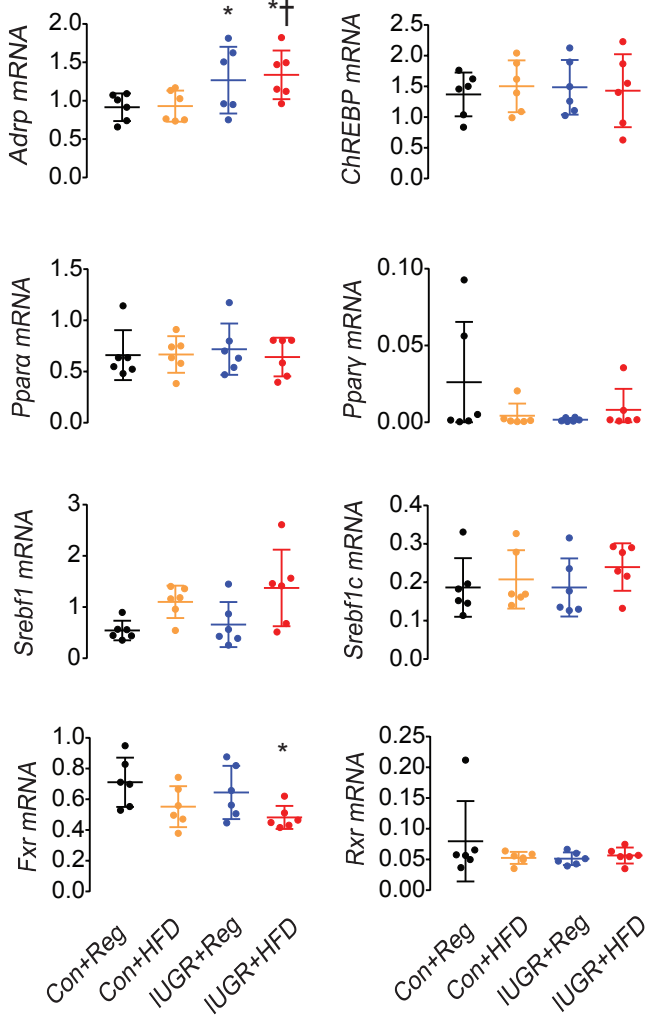

# Females

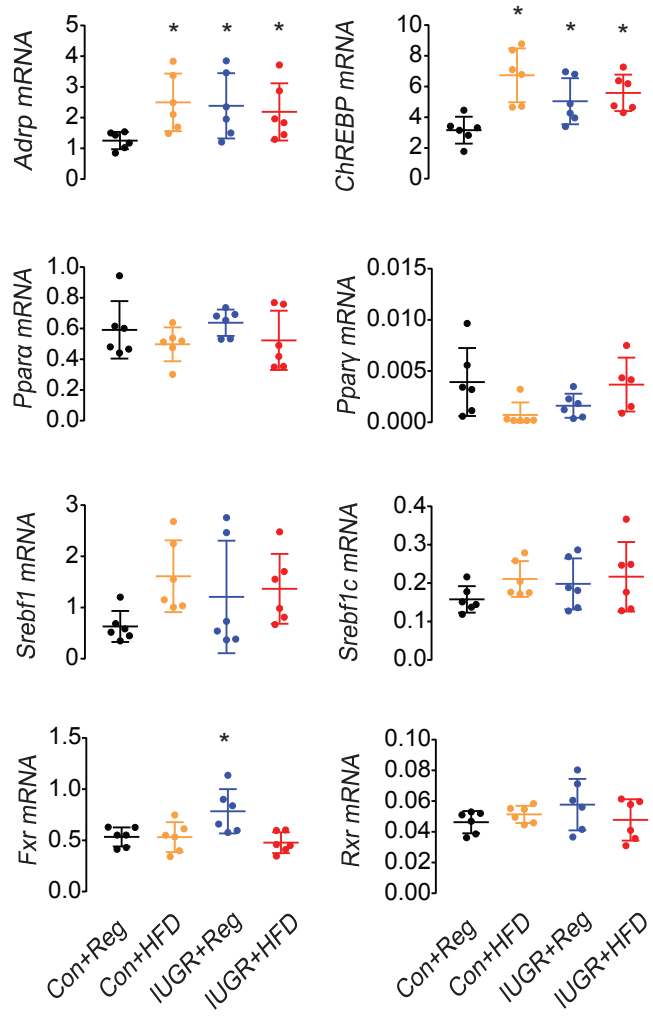

Supplement: FIGURE S2 — IUGR+Reg and IUGR+HFD male and female rats had increased Adrp mRNA, and IUGR+Reg and IUGR+HFD female rats had increased ChREBP mRNA. There were no differences in Pparα, Pparγ, Srebf1, Srebf1c, Fxr, or Rxr mRNA levels. Data shown as scatter plots of individual rats with mean ± SD for n = 6 rats per sex, per intrauterine environment, each from separate litters. Data from male rats is shown on the left of the figure, and from female rats is shown on the right. Groups are denoted as follows: Con+Reg data are shown in black, Con+HFD data are shown in yellow, IUGR+Reg data are shown in blue, and IUGR+HFD data are shown in red, with group names listed below the graphs on the bottom of the figure. [file Image_2.PDF]

## Males

---

Con+Reg

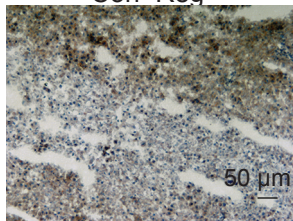

IUGR+Reg

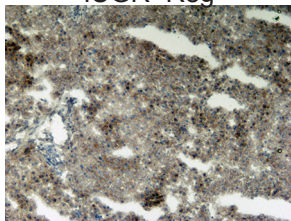

Con+HFD

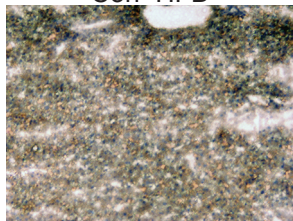

IUGR+HFD

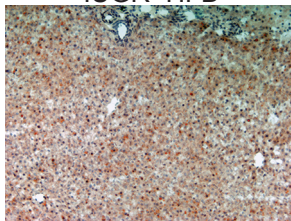

## Females

---

Con+Reg

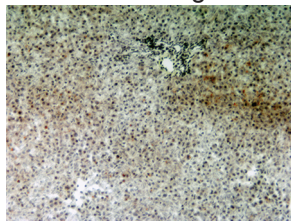

IUGR+Reg

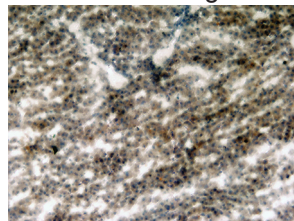

Con+HFD

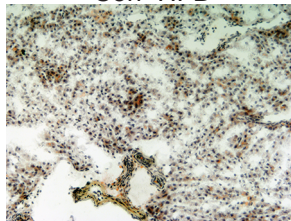

IUGR+HFD

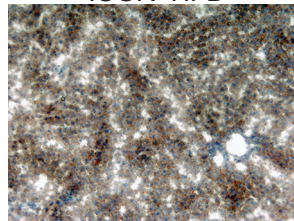

Supplement: FIGURE S3 — Images of left lobe of the liver stained with Oil Red O and hematoxylin. Bar represents 50 μm. [file Image_3.PDF]
